# CWPO Degradation of Methyl Orange at Circumneutral pH: Multi-Response Statistical Optimization, Main Intermediates and by-Products

**DOI:** 10.3389/fchem.2019.00772

**Published:** 2019-11-14

**Authors:** Viviana A. Gómez-Obando, Ana-M. García-Mora, Jessica S. Basante, Arsenio Hidalgo, Luis-Alejandro Galeano

**Affiliations:** ^1^Laboratorio de Catálisis, Grupo de Investigación en Materiales Funcionales y Catálisis (GIMFC), Departamento de Química, Universidad de Nariño, Pasto, Colombia; ^2^Departamento de Matemáticas y Estadística, Centro de Estudios y Asesoría en Estadística (CEASE), Universidad de Nariño, Pasto, Colombia

**Keywords:** methyl orange, Al/Fe-PILC, response surface methodology, statistical optimization, catalytic wet peroxide oxidation

## Abstract

The catalytic wet peroxide oxidation (CWPO) of the industrial azo-dye methyl orange (MO) activated by an Al/Fe-pillared clay catalyst was optimized by the Response-Surface Methodology (RSM). Three sequential sets of factorial 2^k^ central composite experiments were required for the full optimization of the process; catalyst loading and stoichiometric dose of hydrogen peroxide were the experimental factors studied through different times of reaction by means of all, Dissolved Organic Carbon (DOC) removal, Total Nitrogen (TN) removal, reacted fraction of hydrogen peroxide, and decolorization as experimental responses to be maximized. The resulting single-response RSM optimums were combined in a multi-response Desirability function ruling out the differential effect of adsorption on the catalyst's surface by defining all responses per gram of clay catalyst. Former two statistical sets of experiments (DOE-1 and DOE-2) showed the CWPO degradation of MO to get favored at increasing both catalyst loading and time of reaction (up to 180 min). Afterwards, third final design of experiments (DOE-3) displayed 75% of DOC removal, 78% of TN removal, 97% of reacted H_2_O_2_, and 95% of decolorization by using a catalyst loading of 5.0 g/L of Al/Fe-PILC together with just 50% of the stoichiometric amount of H_2_O_2_. The multi-response optimum conditions based on the Desirability function showed excellent fitting explaining at least 99.3% of the optimal overall responses at 95% of confidence. A further analysis revealed that no one of the non-controllable variables under real conditions of industrial wastewater treatment (covariates): starting total organic carbon (TOC) (2.0–20 mg/L), temperature (5.0–25°C) or circumneutral pH (6.0–9.0), exhibited statistically significant effect (*P* > 0.05), suggesting the system to be almost insensitive against them within studied range of close to ambient conditions in the tropic. Finally, HPLC/PDA and GC/FID measurements identified phenol, cyclohexa-2,5-diene-1,4-dione, phenylamine, *N*-methylaniline and *N,N*-dimethylaniline in very low concentrations as main intermediates in the CWPO degradation of MO, which nevertheless disappeared over 90 min of treatment. Meanwhile, 4-aminobenzenesulfonic and oxalic acids were recorded as unique by-products at final time of reaction, but both of them fairly less toxic than the starting azo-dye.

## Introduction

In recent decades, the high growth of unacceptable levels of polluting substances in water has become a danger to the lives of human beings. One of the main problems presently faced is increasing amounts of natural organic matter (NOM) in surface and groundwater. NOM is a complex mixture of aliphatic and aromatic compounds, characterized by their fluctuating amounts in water and variable molecular and chemical properties (Sillanpää et al., 2018). NOM is the result of vegetable remains, decomposing animals, and soil runoff. NOM is mainly made up of carbon and organic nitrogen with a (C/N) ratio of 15 to 50 in many freshwater systems (Reemtsma, 2009). The average total nitrogen in the Colombian rivers is 65 mg N/L, 43.4 mg N/L of which is organic nitrogen and 21.6 mg N/L ammonia nitrogen (Gutiérrez, 2011). These high nitrogen contents, present mainly in the hydrophobic fraction of NOM (humic and fulvic acids) represent one of the biggest challenges faced through the elimination of natural organic compounds from surface waters.

Given that the NOM structure is quite complex, with a significant contribution of phenols and aromatic amines (Goslan et al., 2017), azo dyes such as methyl orange (MO) (Table 1) can serve as a molecular model allowing to anticipate the behavior of the nitrogenous fraction present in dissolved NOM. The MO, besides containing nitrogen (the azo group N = N and the aromatic amine), also contains sulfur and aromaticity, which gives it an intense coloration, toxicity (mutagenesis and carcinogenesis), high chemical stability and low biodegradability. For this reason, it has been used as a model molecule in several studies for the treatment of residual aqueous effluents (Galeano et al., 2010; Panda et al., 2011; Youssef et al., 2016; Liu et al., 2018).

**Table 1:** Some physicochemical properties of the azo-dye methyl orange.

**Characteristic**	**Methyl orange**
Structure	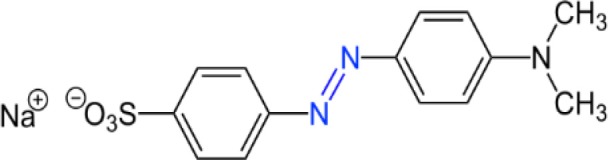
C.I.^a^ name	Methyl orange
C.I.^a^ number	13025
Chemical formula	C_14_H_14_N_3_NaO_3_S
Molecular weight (g/mol)	327.34
p*K*_a_	p*K*_a1_ = 3.4; p*K*_a2_ = 6.1 (de Oliveira et al., 2006)

a *C.I, Color Index*.

The NOM removal in the production of potable water is becoming more and more a difficult challenge due to the increasing concentrations of NOM in surface water along the past few decades as well as because of its variable composition. In order to complete such a goal, it is necessary to develop more efficient and versatile treatment technologies.

Advanced Oxidation Technologies (AOTs) are based on the production of hydroxyl radicals (HO^**•**^) and other Reactive Oxygen Species (ROS) such as the hydroperoxyl radical (${\text{HO}}_{2}^{•}$), its conjugate base, the superoxide anion (${\text{O}}_{2}^{–•}$) and organic peroxyradicals (R-OO^**•**^). These substances almost indistinctly attack all organic molecules present in water (Ormad et al., 2006; He et al., 2016), and can degrade NOM to CO_2_ and water, or convert it to more biodegradable compounds such as aldehydes and carboxylic acids. One of the most promising AOTs is the Catalytic Wet Peroxide Oxidation (CWPO), a heterogeneous Fenton-like process which may use Al/Fe-pillared clays as catalyst, solids exhibiting high chemical stability and activity transforming hydrogen peroxide to the ROS. In the standard Fenton reaction catalyzed by ${\text{Fe}}_{\left(\text{aq}.\right)}^{2+}$ two hydroxyl radicals (HO) are generated by the homolytic breakdown of the O–O bond in the hydrogen peroxide molecule, but when the reaction is started by ${\text{Fe}}_{\left(\text{aq}.\right)}^{3+}$ the mechanism is known as the Fenton-like process, where the first step involves the formation of the hydroperoxyl radical (HO_2_) alongside with the *in–situ* regeneration of the ferrous cation. The mechanism of the Fenton-like process has been described in depth elsewhere (Galeano et al., 2014). This process has displayed numerous advantages over the homogeneous Fenton process, including: the absence of sludge at the end of the process, easy separation of the catalyst from the medium with the possibility of reusing it again, and pH ranges close to neutrality. In similar studies conducted using low pH (between 3.0 and 4.0) (Queirós et al., 2015; Liu et al., 2018), higher operational costs of the process were recorded.

Several parameters influence the practical application of the CWPO reaction, such as concentration of the Fe-bearing catalyst, concentration of H_2_O_2_ (oxidizing agent), pH of reaction, initial concentration of pollutants (for instance, in terms of TOC: Total Organic Carbon and TN: Total Nitrogen), temperature, among others. Taking into account that temperature, pollutant concentration, and pH are variables that could not be controlled when implementing the technology at pilot or real scale, it would be necessary for the previous process optimization to be carried out under variable conditions of the input stream, whereas ensuring economic feasibility. In this sense, the efficiency of TOC removal with the CWPO technology has shown to be strongly influenced by the dose of H_2_O_2_ and particularly by the relationships between (H_2_O_2_/Fe), (H_2_O_2_/contaminant), and (Fe/contaminant) (Soon and Hameed, 2011). The finding of such optimal relationships may allow to elucidate the highest efficiency of generation of hydroxyl radicals and other highly oxidizing ROS from the accurate combination of hydrogen peroxide/catalyst/pollutant, minimizing parasitic reactions that consume H_2_O_2_ (Rueda Márquez et al., 2018).

The response surface methodology can be used to both statistically optimize a process and the prediction of the interaction between the variables thereof, reducing the number of experiments required and therefore the associated time and costs of the optimization process (Bezerra et al., 2008). The RSM has been applied in numerous studies to optimize the treatment of textile dyes in aqueous effluents along the past few years (Azami et al., 2013; Chen et al., 2014; Mousavi and Nazari, 2017). However, in the literature, as long as we know no scientific work has been published related to the application of RSM in the treatment of molecules that simulate the behavior of nitrogen in NOM with Al/Fe-PILC catalysts through the application of the CWPO technology, nor has hydrogen peroxide consumption been used as main experimental factor, governing the overall process efficiency.

Accordingly, this work was devoted to optimize the Al/Fe-PILC catalyzed CWPO degradation of MO by means of the multivariate and multi-response RSM. The effect of the main reaction variables (concentration of catalyst and dose of hydrogen peroxide) on the MO mineralization were determined from the Dissolved Organic Carbon (DOC) and Total Nitrogen (TN) removals, decolorization, and reacted fraction of the added hydrogen peroxide. For such a purpose it has been employed a Central Composite Design (CCD) of experiments, reported as one of the best strategies in statistical process optimization (Arslan-Alaton et al., 2009; Azami et al., 2013; Chen et al., 2014). Finally, the main intermediates and by-products of the CWPO degradation of MO have been identified and quantified by both HPLC/PDA and GC/FID in order to establish if the adopted strategy of treatment do not produce either intermediates or final by-products with levels of toxicity comparable, or even superior, to that of the starting, targeted azo-dye.

## Experimental Section

### Al/Fe-PILC Clay Catalyst

In order to optimize the main operating parameters of the CWPO in the degradation of MO at lab scale, three statistical designs of the catalytic experiments (DOE) were executed using an Al/Fe-PILC clay catalyst prepared according to an adapted method formerly reported (Galeano et al., 2012). The catalyst was prepared from a Colombian natural bentonite refined by particle size and denoted C2R. It was modified with Al and Fe using an auto-hydrolysis method with metallic aluminum followed by a pillaring procedure elsewhere available (Muñoz et al., 2017, 2018) with next final characteristics: nominal Atomic Metal Ratio of iron (AMR_Fe_) = 5.0%; Total Metal Concentration (TMC) = 5.73 mol/L; molar (Al^3+^/Al^0^) ratio = 14/86 in the intercalating solution; calcination at 400°C; final content of incorporated Fe (considered active for this catalytic application) = 0.62 wt. %. The final modified material is henceforth labeled as C2R-PILC. The physicochemical properties of both the starting clay and the final Al/Fe-PILC clay catalyst are summarized in Table S1.

### Statistical Design of Experiments

Response surface methodology was used to optimize the main operating parameters of the CWPO technology in the degradation of MO. Three sequential designs of experiments (DOE-1, DOE-2, and DOE-3) were formulated and executed. In this work the results obtained from DOE-3 set of experiments will be more specifically analyzed as it proved to be the one that resulted in the highest (MO/active Fe/H_2_O_2_) ratio optimization. DOE-1 consisted of a 2^3^ central composite design (2 levels and 3 factors) with 9 central points, 2 axial points (orthogonal and rotatable), with a final multi-response analysis based on the Desirability function. The experimental factors used in the altogether 23 catalytic tests and their chosen experimental ranges were: dose of H_2_O_2_ [(H_2_O_2_)d] = 50–150% of the stoichiometric amount required for full mineralization; concentration of Al/Fe-PILC catalyst [C2R-PILC] = 0.34–1.21 g/L; and reaction time = 60–120 min (which coincided with the time of addition of the hydrogen peroxide dosing on the reacting mixture). In addition, three non-controllable variables were considered: initial pH (recorded but not controlled during the catalytic test), initial TOC concentration and temperature; these variables were simulated using the Montecarlo technique, assuming both normal, and Cauchy distributions, according to the data recorded during a preliminary pilot sampling over 113 samples widely distributed in mountain and coast regions of Nariño province, Colombia. The temperature was recorded between 1.0 and 35.0°C, N distribution (18; 5.67); organic carbon concentration within a range of 0–30 mg TOC/L, Normal distribution (15.2; 5); and the pH variable was simulated with a Cauchy distribution: Mode = 7.03, scale = 0.15. The targeted goal of the multi-response optimization was to maximize following responses at the same time: DOC removal, TN removal, fraction reacted of H_2_O_2_, and MO decolourization, all expressed in % (Table S2).

Subsequently, the DOE-2 design consisted of a 2^2^ factorial central composite set of experiments alongside 4 central points, 2 axial and rotatable points. 10 catalytic tests were then assessed from 2 experimental factors: (i) [C2R-PILC] = 0.5–6.0 g/L and (ii) (H_2_O_2_)d = 25–100%. In this case, the reaction time was set constant at 180 min, based on the observation made in DOE-1 set of experiments raising that an increase in the reaction time always led to greater efficiency. For the statistical analysis, it was found useful to express all response variables divided by the mass of catalyst used in each test in order to eliminate the influence of simple pollutant's adsorption on the statistical optimization, which of course is directly related to the amount of catalyst used. The non-controllable variables were randomized within the following, narrower ranges, in comparison with DOE-1 set of experiments: pH = 6.0–9.0, starting DOC concentration = 2.0–20 mg/L and temperature = 5.0–25°C, then allowing for the statistical analysis to be made in terms of the influence of each factor within such a set of ambient conditions (Table S3).

Finally, the DOE-3 arrange of experiments consisted of a 2^2^ response surface design. 10 experiments were conducted in which the catalyst amount varied within the range 6.50–13.5 g/L and the dose of H_2_O_2_ between 53.7 and 71.3 stoichiometric % (Table 2), in order to establish the typical curved zone within which the optimal performance can be found. The starting values of non-controllable variables pH, TOC and temperature of input water (covariates) in this set of experiments are displayed at Table 3. Once every range was defined, it was randomized taking into account a normal distribution. The optimization goal was simultaneous maximization of Decolourization (466 nm), TN removal, Reacted hydrogen peroxide, and DOC mineralization. The catalytic essays for this final design of experiments were carried out using recycled C2R-PILC catalyst from DOE-2 set of experiments (reused catalyst); to do this so, the catalyst was separated by filtration after conducting every experiment of the previous design and then washed with abundant type-I water under gentle stirring, followed by final drying at 60°C.

**Table 2:** Final statistical design of experiments (DOE-3) used to optimize main operating parameters of the CWPO degradation of MO under close to ambient temperatures and circumneutral pH.

**Experimental variables**	**Units**	**Level lower (-1)**	**Level upper (+1)**	**Central**	**Axial points**
**(H**_**2**_**O**_**2**_**)d**	% Stoichiometric	53.7	71.3	62.5	50–75
**[C2R-PILC]**	g/L	6.5	13.5	10	5–15
**Reaction time (tr)**	180 min				
**COVARIATES**					
**Starting MO Concentration [MO]**_**i**_	DOC (mg C/L)	2.0–20	–	–	
**Temperature of reaction (T**_**r**_**)**	°C	5.0–25	–	–	
**pH**	–	6.0–9.0	–	–	

**Table 3:** Levels of covariates used in third statistical design of experiments (DOE-3) for the CWPO degradation of MO in semi-batch reactor.

**Run**	**[DOC]_**I**_ (mg C/L)**	**pH_**i**_**	**Temperature (^°^C)**
1	12.0	7.2	22.6
2	7.60	8.9	12.3
3	10.7	6.7	6.10
4	9.00	7.2	10.0
5	11.5	7.5	15.1
6	14.6	6.7	21.0
7	18.6	7.1	25.6
8	5.80	7.0	17.6
9	14.9	7.0	23.3
10	10.5	6.9	19.2

### Catalytic Experiments

The catalytic experiments were carried out in a 1.5 L semibatch (Pyrex^®^, glass reactor equipped with a jacket for temperature control by recirculating a thermostatic bath) The reactor had five inputs: (i) to introduce the mechanical stirrer ensuring a homogeneous mixture between the catalyst and the contaminants in the reaction medium; (ii) to enable hydrogen peroxide addition by means of a peristaltic pump feeding the H_2_O_2_ solution under controlled flow; (iii) pH electrode; (iv) air inlet providing constant bubbling directly through the reaction suspension maintaining saturated the liquid phase, and thus avoiding CO_2_ accumulation; and (v) periodical sampling of solution during the reaction.

The C2R-PILC catalyst was evaluated in the catalytic oxidation of methyl orange (MO) in a dilute aqueous medium. The tests were carried out at atmospheric pressure (0.76 atm) and different temperatures (Table 2), according to the next procedure: the powdered catalyst was placed in contact with 500 mL of MO solution in the semi-batch reactor under mechanical stirring (600 rpm). Different concentrations of hydrogen peroxide were tested in order to provide different fractions in comparison with the theoretical dosing necessary for the stoichiometric, full mineralization of the MO content according to Equation (1) (Galeano et al., 2010):

(1) $$\begin{array}{l} {\text{C}}_{14}{\text{H}}_{14}{\text{N}}_{3}{\text{O}}_{3}\text{SNa }+\;43{\text{H}}_{2}{\text{O}}_{2}\to 14\text{C}{\text{O}}_{2}+\;3\text{HN}{\text{O}}_{3}\\ +\text{ NaHS}{\text{O}}_{4}\;+\;48{\text{H}}_{2}\text{O} \end{array}$$

The initial pH was adjusted according to the randomized value (Table 2) and the air bubbling was maintained at ~2 L/h. Addition of 100 mL of the H_2_O_2_ solution prepared in targeted concentration from the commercial reagent (Chemi, 50% w/w) just started after 30 min (15 min for DOE-1) of stirring and air bubbling of the catalyst suspended in the liquid medium (equilibrium period) at constant flow rate throughout 180 min (60–120 min for DOE-1 experiments); samples (25 mL each) were taken along every reaction time. Once finished the addition of hydrogen peroxide the reaction was still recorded by means of an extra period of 30 min (15 min for DOE-1), so that the remaining H_2_O_2_ may react in greater extent. Hence, the total time of every catalytic test was 240 min (for DOE-2 and DOE-3). Each sample was immediately microfiltered (ϕ = 0.45 μm, PVDF filters) after being collected and reserved for following analyses: (i) colorimetric (466 nm), (ii) remaining concentration of free peroxide, (iii) Dissolved Organic Carbon (DOC) and (iv) Total Nitrogen (TN); besides, (v) HPLC/PDA, and (*vi*) GC/FID were further measured in the samples taken from a couple of catalytic experiments developed under the set of optimal conditions released from the multi-response statistical optimization. At the end of each test the catalyst was recovered by vacuum filtration and leached Fe concentration determined through AAS according to Standard Method SM 3030 H.

### Analytical Techniques

#### Decolourization of Methyl Orange Solutions

To determine the MO concentration at different reaction times, 0.5 mL of phosphate buffer was added to each sample at pH = 7.0 (to avoid bathochromic or hypsochromic shifts). The samples were then measured at λ = 466 nm in a Shimadzu UV-2600 spectrophotometer in a range of 200 to 700 nm (linear range: 0–10 mg MO/L, r^2^ = 0.999; detection limit = 0.101 mg MO/L; quantification limit = 0.346 mg MO/L). The percentage of MO decolourization was calculated from Equation (2), where c_0_ and c_t_ represent MO concentrations at initial and *t* times of reaction, respectively:

(2) $$Decolourization\;\left(\%\right)=\frac{C_{0}-C_{t}}{C_{0}}\;x\;100$$

#### Dissolved Organic Carbon (DOC) and Total Nitrogen (TN)

DOC analysis was determined after full removal of particulate solids by filtration (ϕ = 0.45 μm, PVDF filters). The MO mineralization was monitored with a Shimadzu TOC-L CPH Analyzer according to standard methodology employing potassium acid phthalate (Panreac, 99.95%), sodium carbonate (Sigma-Aldrich, 99%), sodium bicarbonate (Sigma-Aldrich, 99.5%), and a sodium nitrate (Sigma-Aldrich, 99%) as calibration standards (detection limit = 0.518 mg C/L; quantification limit = 1.121 mg C/L). All the carbon present in the sample was converted to CO_2_ at 680°C through a catalytic furnace and dragged to a non-dispersive infrared detector. It does not differentiate carbon from different chemical compounds, but it does differentiate between inorganic and organic carbon, since the former one can be volatilized in advance by acidification of the solution. The DOC (mg C/L) was finally calculated as the difference of total carbon and inorganic carbon contents. Meanwhile, the TN content was converted to NO in the catalytic furnace set to 720°C and then dragged to a chemiluminescence detector (mg N/L). DOC and TN measurements were both performed from three replicates each using 16 mL samples for both, whose free content of peroxide was previously deactivated by addition of 0.5 mol/L of sodium sulfite (Panreac, 98%). DOC and TN removals were either defined as shown at Equation (3):

(3) $$DOC/TN\;\left(\%\right)=\frac{C_{0}-C_{t}}{C_{0}}x\;100$$

Where *c*_0_ and *c*_*t*_ represent either DOC or TN concentrations at the beginning of the experiment and after a *t* time of reaction, respectively.

#### Iodometric Determination of Remaining Content of Free Hydrogen Peroxide

Throughout each catalytic tests, absorbance of the yellow solution formed by the reaction between the remaining H_2_O_2_ and potassium iodide at λ = 361 nm was measured in a Shimadzu UV2600 spectrophotometer (Klassen et al., 1994). For this, 0.2 mL of 0.01 mol/L ammonium molybdate (Merck, 81–83%) were added on 0.5 mL of sample, along with 0.3 mL of 1.0 mol/L sulfuric acid (Panreac, 98%) and 2.0 mL of potassium iodide 0.1 mol/L (Carlo Erba, 99.5%): linear range = 0–15.0 mg/L, r^2^ = 0.999, detection limit 0.01 mg/L; quantification limit = 0.06 mg/L. The reacted fraction of hydrogen peroxide (Equation 7) was calculated from the total volume of the reaction mixture (*V total MO*) at the *t* time, the measured concentration of hydrogen peroxide at each reaction time ([_*H*_2_*O*_2_*rem*]*t*_), the added volume of H_2_O_2_ (*V total add*_*H*_2_*O*_2__) and the other parameters indicated in Equations (4–7).

(4) $$\begin{array}{l} {\left(\text{mmol }{\text{H}}_{2}{\text{O}}_{2\;}\text{rem}\right)}_{\text{t}}={\left(\text{V total MO}\right)}_{\text{t}}*{\left[{\text{H}}_{2}{\text{O}}_{2}\text{ rem}\right]}_{\text{t}} \end{array}$$

(5) $$\begin{array}{l} {\left(\text{mmol }{\text{H}}_{2}{\text{O}}_{2\;}\text{total added}\right)}_{\text{t}}={\left(\text{V total ad}{\text{d}}_{{\text{H}}_{2}}{\text{O}}_{2}\right)}_{\text{t}}*{\left[{\text{H}}_{2}{\text{O}}_{2}\right]}_{\text{i}} \end{array}$$

(6) $$\begin{array}{lll} {\left(\text{mmol }{\text{H}}_{2}{\text{O}}_{2\;}\text{reacted}\right)}_{\text{t}} & = & {\left(\text{mmol }{\text{H}}_{2}{\text{O}}_{2\;}\text{total added}\right)}_{\text{t}}\\ \; & \; & -{\left(\text{mmol }{\text{H}}_{2}{\text{O}}_{2}\text{ rem}\right)}_{\text{t}} \end{array}$$

(7) $$\begin{array}{l} {\text{H}}_{2}{\text{O}}_{2\;}\text{ reacted}\;(\%)=\;\frac{{\left(\text{mmol}\;{\text{H}}_{2}{\text{O}}_{2}\text{ reacted}\right)}_{\text{t}}}{{\left(\text{mmol}\;{\text{H}}_{2}{\text{O}}_{2}\;\text{total}\;\text{added}\right)}_{\text{t}}}*100 \end{array}$$

### Efficiency of H_2_O_2_ Consumption and Selectivity to Mineralization in the CWPO Degradation of MO

The selectivity toward mineralization *(*σ*)* was defined according to Equation (10) by the ratio between experimental consumed DOC (Equation 8) and theoretical DOC if the MO consumption would fully correspond to mineralization as measured by visible spectroscopy (Equation 9):

(8) $$\text{Consumed DOC }(\text{mg/L})=\left[DOC_{i}\right]-[DOC_{f}]$$

(9) $$\text{Theoretical DOC }(\text{mg/L})={\left[DOC_{i}\right]}_{MO}-{\left[DOC_{f}\right]}_{MO}$$

(10) $$\sigma =\frac{\;Consumed\;DOC\;(mg/L)}{Theoretical\;DOC\;(mg/L)}*100$$

Efficiencies of hydrogen peroxide consumption previously defined by Zazo et al. (2010) and Inchaurrondo et al. (2012) were adopted in this study (Equations 13 and 14), where η is the ratio between DOC and hydrogen peroxide consumed in the final reaction time (Equation 11), and ε is the amount of DOC consumed per unit mass (g) of hydrogen peroxide fed (Equation 12).

(11) $$\begin{array}{l} H_{2}O_{2}\;consumed\;(\frac{g}{L})=\\ \text{​​}\frac{\;Stoichiometric\;H_{2}O_{2}\;added\;\left(g\right)*(H_{2}O_{2}\;reacted)t_{f}\;(\%)}{Volume\;of\;MO\;added\;in\;the\;reactor(L)} \end{array}$$

(12) $$H_{2}O_{2}\;fed\;\left(\frac{g}{L}\right)\;=\;\frac{\;H_{2}O_{2\;added}\;in\;the\;reactor\;(g)}{MO_{\;added}\;in\;the\;reactor\;(L)}$$

(13) $$\eta =\frac{\;Consumed\;DOC\;(mg)}{H_{2}O_{2\;}consumed\;(g)}$$

(14) $$\varepsilon =\frac{\;Consumed\;DOC\;(mg)}{H_{2}O_{2\;}fed\;(g)}$$

### Identification and Quantification of the Main Intermediaries and by-Products of the CWPO Degradation of MO

#### Chromatographic Analysis Through HPLC/PDA

The analysis of the aromatic intermediates and short chain carboxylic acids obtained during the MO degradation was carried out in a Shimadzu Prominence HPLC equipment. 3.0 mL of methanol (HPLC/PDA grade, Panreac) were added on 7.0 mL of sample collected from the catalytic test developed under optimal conditions of reaction, then filtered (ϕ = 0.45 μm, PVDF filters), and measured. The fractions were separated in a Premier C18 column (5.0 μm × 4.6 mm × 250 mm, Shimadzu) at 30°C. Injection volume was 30 μL and flow 0.8 mL/min, using a gradient method (A/B) with methanol (A) and water acidified with phosphoric acid at pH 2.3 (B) as mobile phases (0–5 min, 0/100 v/v; 10 min, 10/90 v/v; 15 min, 40/60 v/v; 20 min, 60/40 v/v; and 30 min 70/30 v/v). Following compounds were used to calculate response factors of targeted analytes: methyl orange (Sigma-Aldrich, 85%), 4-aminobenzenesulfonic acid (Sigma-Aldrich, 99%), Propanedioic acid (Sigma-Aldrich, 99%), maleic acid (Sigma-Aldrich, ≥99%), Butanedioic acid (Merck, 99.99%), ethanoic acid (Panreac, 99.8%), methanoic acid (Carlo Erba, 85%), oxalic acid (Alfa Aesar, 99%), benzenesulfonic acid (Sigma-Aldrich, 98%), benzene-1,4-diol (Panreac, 99%), Cyclohexa-2,5-diene-1,4-dione (Fluka, ≥99.5%), phenol (Sigma-Aldrich, 99%), *p*-nitrophenol (Alfa Aesar, 99%), and Benzene-1,2-diol (Alfa Aesar, 99%).

#### Chromatographic Analyses Through GC/FID

In order to monitor the volatile intermediates and by-products (mainly, the most concerning aromatic amines), the samples were injected in a Shimadzu GC-2010 gas chromatograph provided with flame ionization detector (FID) and split injection mode; 5.0 mL of sample were analyzed in this case. Three successive extractions were conducted with 3.0 mL portions of ethyl acetate (Panreac, 99.5%); the sample was concentrated in a Heidolph rotaevaporator at 40°C up to 2.0 mL, and then measured. Separations were made under a temperature program by using an PTA-5 Fused Silica capillary column (30 m × 0.53 mm × 1.5 μm from SUPELCO). The GC conditions of separation were: Detector and injector temperature: 220°C, carrier gas He, make up gas N_2_ (40 mL/min), oven temperature program: 130–150°C under a ramp of 1.0°C/min, then 150–180°C under a ramp of 10°C/min, and finally 1 min at 180°C. Following standards were used to calculate response factors: Phenylamine (Mallinckrodt, 99.99%), *N*-methylaniline (Sigma-Aldrich, ≥99%), *N,N*-dimethylaniline (Sigma-Aldrich, 99.57%), N,N-dimethyl-p-phenylenediamine (Alfa Aesar, 96%), 3-Dimethylaminophenol (Alfa Aesar, 97%).

## Results and Discussion

### Preliminary Trials

The results of DOE-1 set of experiments showed as a general trend that with higher concentrations of catalyst and lower doses of H_2_O_2_, the mineralization of methyl orange (MO) increased. It was then established that the concentration of the clay catalyst exerted the most influencing effect on the application of the CWPO technology, at least within the ranges of reaction and environmental conditions here assessed (Figure S1). Subsequently, the results obtained in the second statistical arrange of experiments DOE-2 (Figure S2) showed that the amount of catalyst should be further increased and the H_2_O_2_ dose decreased in order to more closely approach the optimal set of conditions. Thus, a third arrange of experiments DOE-3 was executed implementing such a set of changes defining targeted ranges for the experimental factors. This work presents a more detailed analysis of this last set of experiments and the optimal reaction conditions that were achieved from it. A (H_2_O_2_/Fe) ratio between 0.56 and 4.70 was employed, along with a higher catalyst concentration (5.0–15 g/L) and a dose of H_2_O_2_ lower to the stoichiometric (50–75%) according to Equation (1), in order to obtain a proper curvature and the optimal point in the multiple-response surface ensuring the highest efficiency for the Al/Fe-PILC catalyzed CWPO degradation of MO in the terms above explained, which enabled both, higher decolourization and mineralization (C and N) as well as the lower cost thanks to the improvement of the reacted fraction of dosed hydrogen peroxide, in other words, yielding the highest possible use of the reagent by the catalytic system.

### Screening of the Third Statistical Arrange of Experiments (DOE-3)

The DOE-3 experimental design enabled the efficient estimation of the quadratic effects and the higher order interactions, as well as the determination of the non-linear nature of the response variables in the experimental design (Table S1). The results obtained are summarized in Table 4 and allowed to establish the following:

- Experiment 10 displayed the highest value in all the responses using low levels (Table 4) of both H_2_O_2_ dose (53.7%) and catalyst loading (6.5 g/L).
- In addition, it was observed that the molar ratio H_2_O_2_/Fe was 2.0; as later shown, the combination of factors used in this experiment was very close to that one finally resulting optimal, which took the highest advantage from both the quantity of active Fe in the C2R-PILC catalyst and the added amount of hydrogen peroxide.
- In tests 1 and 5, although the levels of catalyst loading and H_2_O_2_ dose were the same, test 5 displayed higher decolourization than test 1 (around 10% higher); it could be due to change in temperature (Table 3): test 1 was made at 22.6°C whereas test 5 at 15.1°C, which suggests that lower temperature favored decolourization of the azo-dye, in contrast to other studies which have shown increased decolourization as the temperature increases (>25°C) (Banković et al., 2012). Moreover, slight differences in [DOC]_i_ and pH_i_ would also partially explain this behavior.
- Experiment 7 showed very low percentage of mineralization of either N or C contents (4.0–5.0%) and a decolourization of 12%. This trial was performed under greater assessed (H_2_O_2_/Fe) ratio as well as under a higher dose of H_2_O_2_ than that used in trial 10. Such a set of low responses and the reacted fraction of peroxide (57%) could be explained by the presence of parasitic reactions caused by the strong excess of H_2_O_2_ with respect to the maximum that could be activated by the amount of active iron present. These reactions include: the direct attack of hydrogen peroxide on the molecule, clearly displaying lower oxidation potential that the one exhibited by hydroxyl radicals, depleting oxidizing efficiency; the recombination of the excess of hydroxyl and other oxidizing radicals, especially under low concentrations of the organic pollutants; and the decomposition of hydrogen peroxide to generate hydroperoxyl radicals promoted by the higher pHs of reaction, exhibiting lower oxidation potential than hydroxyl radicals.
- Trial 8 used the highest amount of catalyst (15 g/L) together the with mid-level peroxide dose (62.5%), which resulted in low mineralization degree (<30%) with the lowest (H_2_O_2_/Fe) molar ratio. Accordingly, it was expected that the degradation increased as the amount of catalyst increased, due to availability of greater concentration of active sites in the reacting mixture. However, too high catalyst loading also showed to cause the HO^∙^ radicals to be consumed by surface Fe^2+^ sites, thus reducing the rate of degradation (Bokare and Choi, 2014; Arshadi et al., 2016).
- In Trials 2 and 6, which used the same amount of catalyst (10 g/L) but different doses of H_2_O_2_ (50 and 75%), it was observed that the lower the amount of H_2_O_2_, the higher the percentage of decolourization and mineralization. This also occurred upon comparison of Trials 7 and 10, where a low level of catalyst loading was used, together with a dose of H_2_O_2_ = 71.3 or 53.7%, respectively.
- Trials 1 and 3 employed the same dose of H_2_O_2_ (62.5%) and different amounts of catalyst (10 and 5 g/L). Greater decolourization and mineralization was achieved using a larger amount of catalyst, as there is a greater number of active sites available for the reaction to occur. Likewise, for trials 4 and 7, where a dose of 71.3% of H_2_O_2_ was used along with quantities of [C2R-PILC] that corresponded to the high and low levels (13.5 and 6.5 g/L), better results were obtained using a larger amount of catalyst.

**Table 4:** Experimental results from third statistical design of experiments (DOE-3) delivering optimized Al/Fe-PILC activated CWPO degradation of MO in semi-batch reactor.

**Run**	**Factor**		**Response**				**(H_**2**_O_**2**_/Fe) (mmol/mmol)**	**[Fe] leached ^**b**^ (mg/L)**	**Fe leached ^**c**^ (%)**
	**(H_**2**_O_**2**_)d** **(% Stoich.)**	**[C2R-PILC] (g/L)**	**Mineralization**		**H_**2**_O_**2**_ reacted** **(%)**	**Dec.^**a**^** **(%)**			
			**DOC removal** **(%)**	**TN removal** **(%)**					
1	62.5	10.0	53	42	92	54	1.73	0.18	0.30
2	50.0	10.0	47	26	87	57	0.88	0.20	0.32
3	62.5	5.00	31	19	64	23	3.08	0.07	0.22
4	71.3	13.5	30	21	83	31	1.10	1.48	1.77
5	62.5	10.0	52	51	95	65	1.66	0.15	0.24
6	75.0	10.0	9	10	88	34	2.52	0.15	0.24
7	71.3	6.50	5	4	57	12	4.70	0.18	0.46
8	62.5	15.0	24	26	69	24	0.56	2.04	2.19
9	53.7	13.5	24	38	68	20	1.37	1.15	1.38
10	53.7	6.50	89	83	100	97	2.00	0.24	0.59

a Decolourization (466 nm).

b Fe leached at final reaction time.

c *Respect to content of active Fe in the clay catalyst = 0.62 wt %*.

Such summarized results from DOE-3 set of experiments developed in the presence of the used catalyst also suggest good chemical stability and reusability of this type of clay catalyst. It can be clearly observed that catalytic activity of the modified layered material was maintained even after its use in the DOE-2 experiments; also, taking into account that the leached Fe did not exceed concentrations in the order of 0.6 mg/L which are not significant activating homogeneous Fenton on this kind of strongly bio-refractory azo dye pollutants. Meanwhile, long-term use also seems feasible, since the full percentage of Fe leached from the catalyst along all experiments was below 2.5% respect to the stabilized content by pillaring of the starting aluminosilicate (Table 4). Leaching was therefore negligible, which confirmed the chemical stability of C2R-PILC under the investigated conditions. The CWPO degradation of MO in the presence of Al/Fe-PILCs prepared as in our study can therefore be considered a predominantly heterogeneous catalytic process that does not get significantly interfered by the classical, homogeneous Fenton degradation. Furthermore, stability of the same clay catalyst here used was studied in depth in our previous work (Ramírez et al., 2018), disregarding either significant Fe leaching, pore blocking or loss of structural features through 32 h of CWPO phenol degradation in a continuous flow reactor. A careful overlook on the summarized comparisons made in Table 4 easily allows concluding that a multi-response optimization of this multivariate process was essential in order to elucidate optimal combination of factors ensuring both best molar ratio (H_2_O_2_/active Fe) and the best MO degradation, through the most effective possible use of the consumed hydrogen peroxide.

### Statistical Optimization of the CWPO Degradation of MO Catalyzed by an Al/Fe-PILC

The statistical analysis was performed in order to determine optimal reaction conditions from DOE-3 arrange of experiments. The RSM yielded a statistical model for each response (Equations 16–19) as a function of: (A) dose of H_2_O_2_ and (B) concentration of the C2R-PILC Al/Fe clay catalyst. Note that every response was compulsory to be expressed by unit mass of solid catalyst (Table S4) in order to eliminate effect of adsorption on the statistical optimization; otherwise, expected and obvious trends would be also obtained by using the statistical approach, that is, maximal values of every experimental factor favoring maximal responses:

(15) $$\text{DOC removal}(\%/g\;Cat.)=94.5042+0.361588A-14.3125B-0.0254068A^{2}+0.228003AB-0.0609805B^{2}$$

(16) $$\text{TN removal}(\%/g\;Cat.)=61.0628+0.734066A-11.0286B-0.0243782A^{2}+0.184821AB-0.0709727B$$

(17) $${\text{H}}_{2}{\text{O}}_{2}\text{ reacted}(\%/g\;Cat.)=96.0178-0.564281A-8.91772B-0.00724175A^{2}+0.131061AB-0.0548777B^{2}$$

(18) $$\text{Decolourization}(\%/g\;Cat.)=142.895-1.54045A-12.2652B-0.0102781A^{2}+0.236282AB-0.189268B^{2}$$

The coefficients of determination (R^2^) displayed by the models for each response (individual optimums) decreased in the following order: ${\text{R}}_{\left(\text{Perox}.\text{react}.\right)}^{2}$ > ${\text{R}}_{\left(\text{DOC removal}\right)}^{2}$ > ${\text{R}}_{\left(\text{Dec}.\right)}^{2}$ >R^2^_(TNremoval)_ (Figure S3). The highest level of fitting for the first two exceeding 90% indicates that the responses satisfactorily correlated with the quadratic models and predict well the consumption of hydrogen peroxide and to a lesser extent the mineralization of DOC and the decolourization in all trials. The good fitting of the statistical models to the experimental results even under increased variability due to the change in the starting covariates (pH, T, and DOC_i_) is a quite promising result, since it shows that it properly works within a set of real, ambient conditions, facilitating a larger scale implementation of the CWPO degradation of MO and probably other colored wastewaters from the textile industry.

The identification of most statistically significant factors affecting the CWPO reaction in the MO degradation were made possible through the Pareto chart for each response (Figure S4); Figures 4A,C demonstrate that the interaction AB (where A: dose of H_2_O_2_ and B: concentration of [C2R-PILC] clay catalyst) was directly proportional to both the DOC removal and the reacted fraction of hydrogen peroxide; thus, an increase in the AB interaction significantly favored the percentage of MO mineralization, whereas increased the percentage of reacted peroxide. It unequivocally shows the optimal set of conditions realized corresponds to those under which the mineralization of DOC primarily obeyed to the catalytic activation of hydrogen peroxide through the Al/Fe-PILC catalyst (the CWPO process), where the side reactions displayed a negligible or just marginal effect. Meanwhile, the concentration of catalyst (B) was inversely proportional to these response variables within the experimental ranges studied.

In Figure S4B, it can be seen that the AB interaction remained being the most influential on the TN removal; however, in the case of this response no statistical significance of any of the factors or their interactions was observed. It suggests that the kinetics of nitrogen mineralization present in the MO molecule is very different from the one by which the DOC removal takes place. MO can be transformed into ${\text{NH}}_{4}^{+}$, ${\text{NO}}_{3}^{-}$ ions as well as in the most desirable N_2_ thanks to the high oxidizing power of the generated radical species. With nitrogen, it is necessary to reach the nitrogen atom in oxidation number (0); likewise, in the case of carbon the mineralization up to CO_2_ requires the C oxidation state (4^+^). Throughout the series of catalytic tests, it was found in some cases that nitrogen mineralization was higher than the carbon mineralization or vice versa; in addition, the intermediary species prevalent in the reaction were not recorded as long as total nitrogen was measured. In this regard, from the application of a photocatalytic processes, it was recently suggested that the attack of HO^•^ on the α-carbon atom of the nitrogen group in organic compounds leads to the formation of ${\text{NH}}_{4}^{+}$ through an amide intermediate followed by an additional hydrolysis (Bamba et al., 2017). It was reported that the direct attack of HO^•^ on the nitrogen atom can produce ${\text{NO}}_{3}^{-}$, whereas the N_2_ is generally produced through the photodegradation of the -N = N- bond; apparently, the mineralization of nitrogen strongly depends on the degree of initial oxidation of this element contained in the pollutant to be degraded (Bamba et al., 2017). In Figures S4C,D, it was observed that the interaction of main factors AB had a positive standardized effect on the reacted H_2_O_2_ and MO decolourization, as the combined increase of the mentioned factors also increased the production of highly oxidizing species and favored the speed of the CWPO reaction. It is therefore confirmed that the (H_2_O_2_/Fe) ratio, directly related to the (AB interaction) is a determining factor in the overall process efficiency.

**Figure 1:**
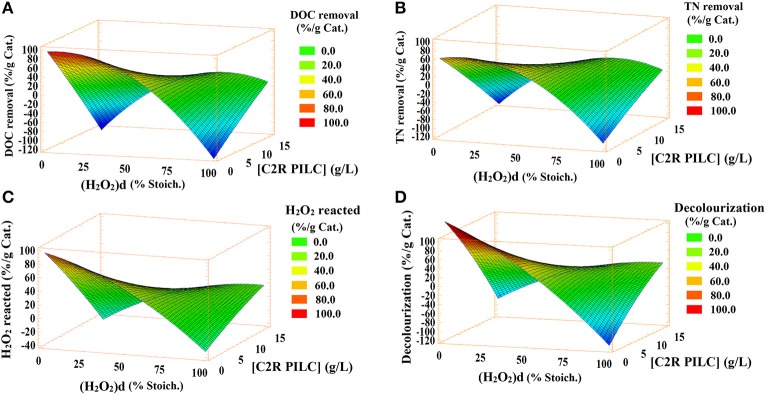
Single-response surfaces: **(A)** DOC removal, **(B)** TN removal, **(C)** H_2_O_2_ reacted, **(D)** Decolorization.

The single-response surface diagrams in Figure 1 allowed to elucidate the general trends about the effects exerted by each factor on the MO degradation. Figures 1A–D show that high percentages of DOC removal, TN removal, and decolourization were favored at lower possible values of the factors.

The Table 5 shows the values of the experimental factors for which the individual optimum of each response was reached and the predicted value for each response as well as the multi-response Desirability function by which the global optimum of the process could be achieved. It is evident that all responses coincided with the lower axial values of factors in the experimental design DOE-3; so it can be raised that the optimal values found within the assessed ranges of the factors in this set of experiments corresponded to the minimum amounts necessary to achieve a significant efficiency in the MO degradation. Moreover, the multi-response Desirability function displayed excellent fitting to individual optimums, predicting 99.32% of the individual responses; according to Table 5, if just a half of the stoichiometric dose of H_2_O_2_ is used along with 5.0 g/L of catalyst, it is possible to achieve the higher MO degradation. On the other hand, the Table 5 displays both the standard error and the coefficient of variation between the replications of the experiment carried out under the optimal set of conditions predicted by the statistical analysis. The low values in both cases allow us to infer good reproducibility of the experimental data here recorded.

**Table 5:** Experimental responses determined under optimal conditions of reaction realized from DOE-3.

**Response**	**(H_**2**_O_**2**_)d (% Stoich. dose)**	**[C2R-PILC] (g cat/L)**	**Predicted optimal response^**a**^ (%/g Cat.)**	**Experimental optimal response^**b**^ (%/g Cat.)**	**SE^**c**^ (%/g Cat.)**	**CV^**d**^ (%)**
DOC removal	50	5.0	33.0	30.2	± 2.6	6.2
TN removal			26.1	32.1	± 1.2	2.6
H_2_O_2_ reacted			36.5	40.2	± 1.0	1.8
Decolorization			33.2	39.6	± 0.9	1.6
**Global desirability (optimum value)**						
0.9932						

a *Optimal values predicted by every statistical model*.

b *Average experimental values from two replicates*.

c *SE, Standard error at 95% of confidence*.

d *CV, Coefficient of variation*.

**Figure 2:**
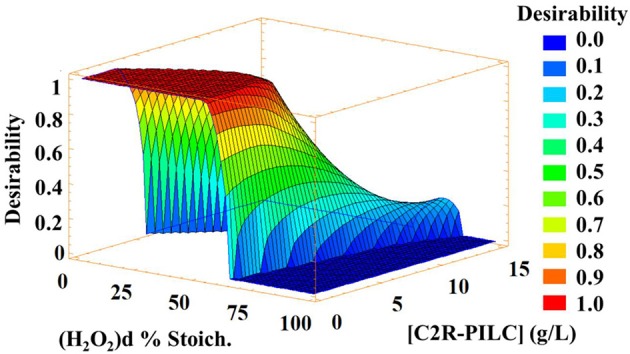
Multi-response surface (Desirability-function). Optimization goal: maximize all DOC removal, TN removal, reacted fraction of H_2_O_2_, and Decolourization.

As said, the fitting of the multi-response Desirability function was excellent, given that this function combined the individual optimum of each response, to yield and overall optimum that represents the best efficiency the CWPO process may exhibit based of the four responses at the same time. In this case, the global optimum practically achieved to replicate the individual optimum values found for each response. In Figure 2, it is observed that maximum Desirability was achieved when the catalyst concentration and H_2_O_2_ dose decreased, which indicate the set of conditions where the best balance between catalytic sites and oxidizing agent was achieved, promoting higher level of formation of the strongly oxidizing radical species.

In the other hand, according to the *p* > 0.05 neither TOC, pH or T exerted a statistically significant effect on any of the evaluated responses (Table 3); it is here remarkable that the range of values studied for these covariates simulated real surface water conditions. Thus, the optimal conditions of reaction here achieved would properly operate at higher scales always that the process works within the ranges of pH, temperature and DOC of the input stream (covariates) yet optimized. Also regarding this issue, it is remarkable that in this study the dosing (against the stoichiometric, theoretical one) instead of the absolute concentration of hydrogen peroxide was optimized, which might allow to tailor in real time the added amount of peroxide on the basis of the input concentration of the contaminant in a continuous flow reactor. It is noteworthy that the overall efficiency of this catalytic process depends on all: catalyst concentration, dose of hydrogen peroxide, but also the input concentration of the contaminant, which is a non-controllable variable in a real application context.

### Efficiency of H_2_O_2_ Consumption and Selectivity to Mineralization in the CWPO Degradation of Methyl Orange

**Figure 3:**
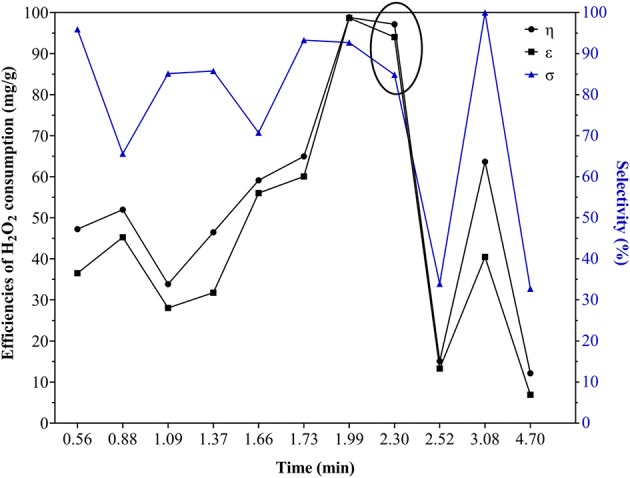
Efficiencies of H_2_O_2_ consumption (η y ε) and selectivity (σ) to mineralization using different ratios (H_2_O_2_/Fe), including the optimal ratio from DOE-3 multi-response optimization (plotted within an ellipse).

Figure 3 shows the selectivity (σ) to mineralization and the efficiencies of this against the fed hydrogen peroxide (ε) and the truly consumed (η), with respect to the (H_2_O_2_/Fe) ratios of each experiment in DOE-3 and the one corresponding to the statistical multi-response optimum. When the (H_2_O_2_/Fe) ratios were 0.56 and 3.08, a close to or even full selectivity to DOC mineralization was achieved, meaning that every intermediate from the methyl orange degradation was also completely mineralized. However, both efficiencies of mineralization respect to the H_2_O_2_ consumption were rather low, in the case of the lower value, probably the amount of peroxide was insufficient to take advantage on the full amount of active iron present on the catalyst's surface, whereas for the higher one it could be ascribed to an increased effect of parasitic reactions affecting peroxide consumption, decreasing the efficiency (Zazo et al., 2010). In opposite way, it was observed that when using an (H_2_O_2_/Fe) ratio of 2.0, σ reached 92.7%, whereas the ε and η efficiencies were similarly very high (98.7 and 98.8%), indicating not only high selectivity toward DOC mineralization but also a highly efficient consumption of the oxidizing agent toward the targeted goal of reaction. This value is close to the optimal ratio obtained from the multiple-response statistical optimization (2.30), demonstrating the utility of the statistical tool in the optimization of this kind of multivariate processes.

### CWPO Degradation of Methyl Orange Under Optimal Conditions of Reaction

**Figure 4:**
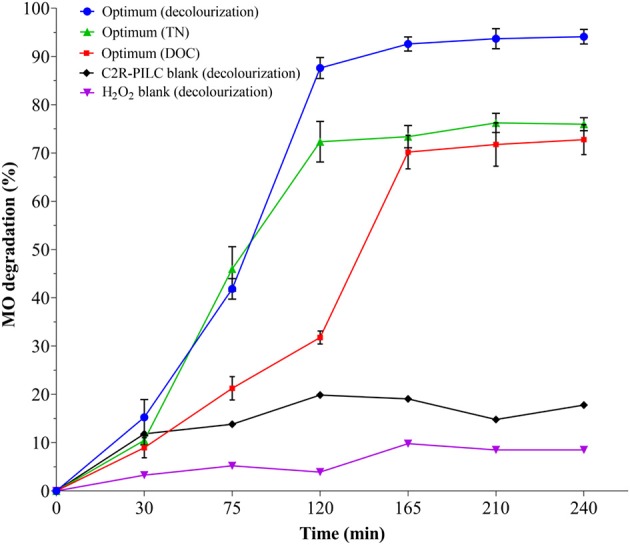
Catalytic behavior in the CWPO degradation of MO activated by an Al/Fe-PILC (C2R-PILC) under optimal conditions of reaction in terms of decolourization, TN and DOC removals: [C2R-PILC] = 5.0 g/L; [MO]_i_ = 0.028 mmol/L equivalent to [DOC]_i_ = 10.0 mg/L, [H_2_O_2_]_added_ = 6.08 mmol/L, V_added_ = 100 mL, H_2_O_2_ stepwise addition = 0.56 mL/min, pH_i_ = 7.0 (it was not adjusted during the reaction), T = 25.0 ± 0.1°C, ambient pressure = 0.76 atm, reaction time = 180 min, tested time = 240 min (included initial 30 min of pre-equilibrium and final 30 min without addition of H_2_O_2_).

**Figure 5:**
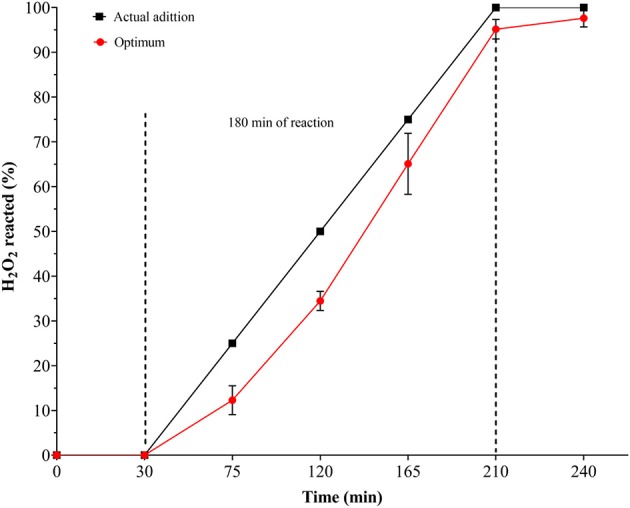
Hydrogen peroxide reacted along the CWPO degradation of MO under optimal conditions. Comparison between actual addition of H_2_O_2_ and reacted H_2_O_2_: [C2R-PILC] = 5.0 g/L, [MO]_i_ = 0.028 mmol/L equivalent TO [DOC]_i_ = 10 mg/L, [H_2_O_2_]_added_ = 6.08 mmol/L, V_added_ = 100 mL; H_2_O_2_ stepwise addition = 0.56 mL/min; pH_i_ = 7.0 (It was not adjusted during the reaction); T = 25.0 ± 0.1°C; ambient pressure = 0.76 atm, reaction time = 180 min, tested time = 240 min (initial 30 min of pre-equilibrium and final 30 min without addition of H_2_O_2_).

Two replicates of a catalytic test were carried out under the optimal conditions revealed by DOE-3, in order to verify fitting of the experimental responses with those expected from the Desirability-based multi-response surface. Figure 4 shows that under optimal conditions of reaction 95% was the maximum decolourization level, together with 75% of DOC and 78% of TN mineralized. In addition, in the presence of just one-half of the stoichiometric dose of hydrogen peroxide the highest efficiency of the process was observed, probably thanks to decreased side reactions scavenging oxidizing radicals. This may indicate, as recently claimed by several authors (Michel et al., 2018; Tarkwa et al., 2019), that there should be an optimal dose of both Fe-catalyst and hydrogen peroxide to achieve the highest conversions, which is being elucidated in this investigation to be (H_2_O_2_/Fe_active_) = 0.15 for the Al/Fe-PILC catalyst. Figure 5 shows that 97% of H_2_O_2_ conversion was also achieved after 180 min of reaction, which clearly explains optimal responses for the heterogeneous Fenton degradation of the azo-dye. In other words, it demonstrates a strong correlation between the peroxide consumption and the best catalytic performance in the pollutant's degradation; since the dosing of the oxidizing agent is far below the theoretical value expected for the stoichiometric transformation by the molecular form of H_2_O_2_, the only way to explain the accomplished high performance of reaction is involving formation of highly oxidizing species including hydroxyl and hydroperoxyl radicals, but probably also other ROS through the catalytic activation of the oxidizing agent.

Since the adsorption blank (C2R-PILC blank) allowed a decolourization not exceeding 20%, the high catalytic response of C2R-PILC was confirmed, and it can be inferred that so high recorded levels of decolourization and mineralization could not be explained by simple adsorption of the contaminant on the catalyst's surface. Likewise, decolourization just in the presence of the oxidizing agent (H_2_O_2_ blank) did not exceed 10%, which indicates as above said that the reaction is preferentially being carried out by the expected mechanism (formation of highly oxidizing radicals) and not as a product of not-activated, direct attack of the hydrogen peroxide on the dye, their intermediates and byproducts.

A comparison of the MO degradation values against those recently reported by the heterogeneous Fenton/CWPO reaction catalyzed by different active solids (namely zeolite, goethite, montmorillonite, activated carbon and nanocomposites (Galeano et al., 2010; Yang et al., 2014; Wang et al., 2015; Liu et al., 2018; Xu et al., 2018) shows that all have been performed under pH conditions below 4.0, still far of the circumneutral conditions here optimized which are featured by most real industrial wastewaters; besides, although full decolourization has been usually achieved, a maximal mineralization of DOC ranging 55–75% was reported (Wang et al., 2015; Liu et al., 2018) in the better of the cases comparable to the 75% here attained under optimal conditions of reaction; moreover, in the study reporting the higher DOC mineralization, around five-fold higher dosing of hydrogen peroxide (268% the stoichiometric) was used. As long as we know no one has reported evolution of the total nitrogen content. Therefore, the optimal conditions here reported clearly enhance the catalytic efficiency exhibited by the CWPO process by decreasing the dosing of hydrogen peroxide, the only one reagent truly consumed throughout.

### Identification and Quantification of the Main Intermediates and Byproducts of the CWPO Oxidation of Methyl Orange

In order to carry out HPLC/PDA and GC/FID recordings of the main reaction intermediaries and by-products, a catalytic test was performed under optimal multi-response conditions but the starting DOC concentration was increased by a factor of around 5 times the one used before for the experimental validation of the statistical multi-response optimum (DOE-3); it was performed this way in order to more safely identify and especially quantify intermediate compounds and reaction byproducts, because when using an initial charge of DOC = 10 mg/L many compounds appeared below the limit of quantification and even no aromatic compounds were identified at all.

**Figure 6:**
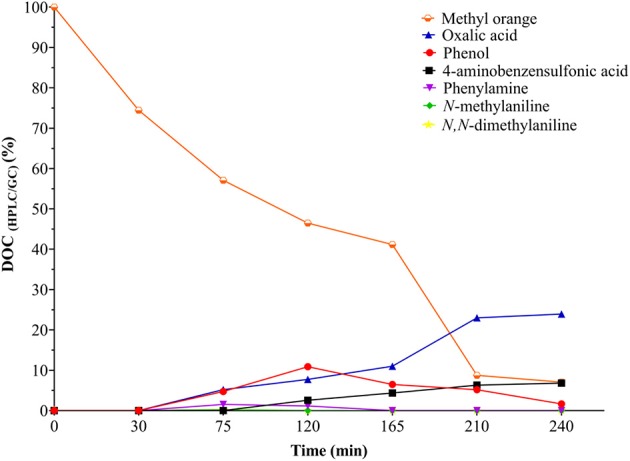
Evolution of intermediates and by-products (measured by HPLC/GC) along the CWPO degradation of MO in terms of dissolved carbon under optimal reaction conditions[MO]_i_ = 0.147 mmol/L equivalent to [DOC]_i_ = 51.3 mg/L, [C2R-PILC] = 5.0 g/L, pH_i_ = 7.0 (it was not adjusted during the reaction); T = 25.0 ± 0.1°C; ambient pressure = 0.76 atm; [H_2_O_2_]_added_ = 31.18 mmol/L, V_added_ = 100 mL H_2_O_2_; stepwise addition = 0.56 mL/min.

**Figure 7:**
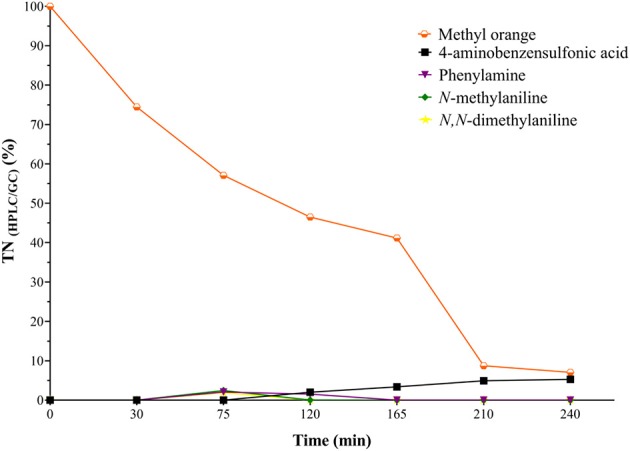
Evolution of intermediates and by-products (measured by HPLC/GC) along the CWPO degradation in terms of total dissolved nitrogen under optimal reaction conditions [MO]_i_ = 0.147 mmol/L equivalent to [TN]_i_ = 10.99 mg/L, [C2R-PILC] = 5.0 g/L, pH_i_ = 7.0 (It was not adjusted during the reaction); T = 25.0 ± 0.1°C; ambient pressure = 0.76 atm, [H_2_O_2_]_added_ = 31.18 mmol/L, V_added_ = 100 mL H_2_O_2_; stepwise addition = 0.56 mL/min.

Figures 6, 7 show the evolution of reaction intermediates and byproducts as a function of the carbon and nitrogen contents measured by HPLC/GC. The material balance with respect to the DOC and TN measurements indicate that after 45 min of reaction the dissolved carbon was mainly due to the formation of phenol and oxalic acid, and the N content was mostly represented in phenylamine, *N*-methylaniline and *N,N*-dimethylaniline, which have been reported as MO oxidation products when using other AOTs (Pillai et al., 2015; He et al., 2016). From 90 min of reaction (120 min of experiment) it was observed that besides the concentrations of phenol and oxalic acid, concentration of sulfanilic acid (4-aminobenzenesulfonic acid) started increasing whereas the concentration of amines began to decrease. Subsequently, as the reaction time further increased, it was observed that the amines completely disappeared and the concentration of phenol decreased until a minimum concentration of 1.11 mg/L (Table S5). These results display the high efficiency of the CWPO process activated by Al/Fe-PILCs in the elimination of refractory and highly dangerous toxic compounds, including the harmful, more highly concerning aromatic amines as intermediates of MO and other azo-dyes. In this sense, it is also remarkable that the CWPO oxidation of the nitrogenous content in the azo-dye more likely led to its mineralization via molecular N_2_ evolved instead than the highly polluting nitrogen oxides; this is somehow easy to hypothesize as long as the starting oxidation states in the aromatic amine moiety (3-) and the azo group (1-) should more easily attain the (0) value for the nitrogen than positive values required to stabilize nitrogen oxides.

**Figure 8:**
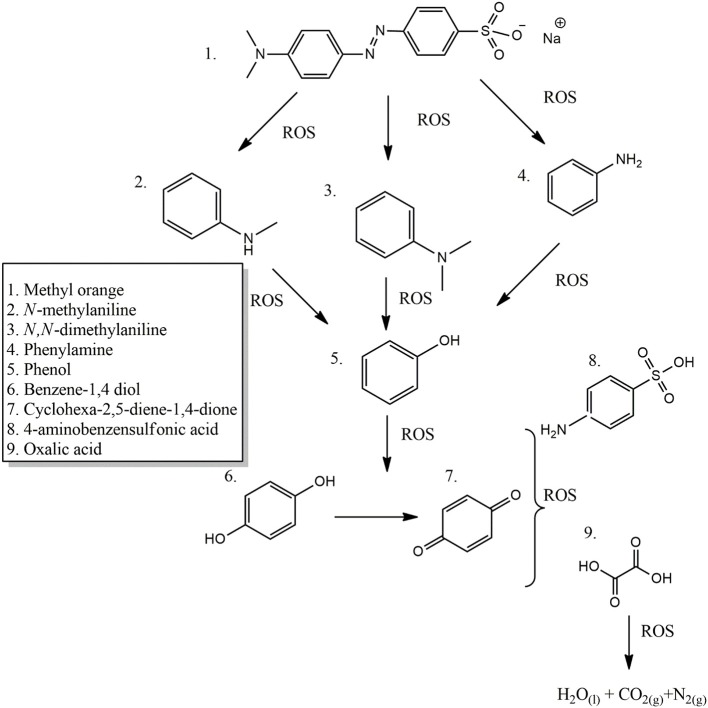
Proposed pathway for the CWPO degradation of MO in the presence of Al/Fe-PILC clay catalyst.

Regarding the oxidation pathway of the MO degradation (Figure 8), it can be inferred that it was carried out in a sequential process that passes through the formation of several intermediates until the production of short chain carboxylic acids, mainly oxalic acid, and 4-aminobenzensulfonic acid which showed to be the only couple of side products truly being accumulated at the final time of reaction, at least within the 180 min here recorded. The MO molecules were probably coordinately adsorbed on the Fe sites in the catalyst's surface by means of σ-type interactions (by using any of the free pairs bearing from the MO molecule) or even π-type from the double bond of the azo group (Banković et al., 2012). The direct attack of different ROS, mainly the radical HO^•^, initially led to the degradation of MO by splitting the azo bond (-N = N-), followed by an electrophilic attack on the azo-dye leading to the hydroxylation of the benzene ring forming phenol; it in turn might follow different paths leading to benzene-1,4-diol, not identified probably because of its rapid oxidation to cyclohexa-2,5-diene-1,4-dione (de Oliveira et al., 2006). Aromatic amines (Phenylamine, *N*-methylaniline, and *N,N*-dimethylaniline) are then formed at early times of reaction but rapidly vanish; finally, short-chain carboxylic acids, mainly oxalic acid and 4-aminobenzenesulfonic acid were generated as final byproducts together with carbon dioxide, molecular nitrogen and water as mineralization products.

## Conclusions

In this work it was achieved the statistical optimization of the CWPO degradation of methyl orange taking into account the most influential factors governing this heterogeneous Fenton-like advanced oxidation process (catalyst loading and H_2_O_2_ dosing), activated by an Al/Fe-PILC clay catalyst. A multi-response approach allowed determining the optimal conditions of said experimental factors simultaneously maximizing the degradation of the contaminant in terms of color elimination, DOC and TN mineralization, through the highest possible utilization of H_2_O_2_ (maximum fraction of reacted peroxide). It successfully elucidated that the maximal efficiency of dissolved carbon mineralization per unit mass of the oxidizing agent was attained at pretty low dosing of H_2_O_2_; indeed, just around half of the amount predicted by the stoichiometric equation for the full oxidation of the starting azo-dye by molecular hydrogen peroxide was enough to achieve it. Besides, it was accomplished within a set of close to ambient conditions of pH (circumneutral), temperature and starting concentration of DOC, narrowly resembling real conditions of polluted surface waters, making cheaper and then more feasible the application of this technological solution in real-scale recovery of polluted water streams.

The most abundant reaction by-products of the azo-dye were the fairly less toxic oxalic and sulfanilic acids. Moreover, main intermediates including the most concerning, harmful aromatic amines, fully disappeared over 90 min of reaction. It unequivocally demonstrated that the CWPO degradation of MO does not produce organic compounds of higher toxicity than the starting dye. Finally, the process under optimal conditions of reaction showed comparable mineralization of the nitrogenous content respect to the DOC removal, seemingly related with formation of strongly inert molecular nitrogen.

## Data Availability Statement

All datasets generated for this study are included in the article/Supplementary Material.

## Author Contributions

VG-O: data acquisition and analysis and draft writing. A-MG-M contribution to the conception of the work. JB: data acquisition. AH: design of the work and statistical analysis of data. L-AG: design of the work, analysis, and chemical interpretation of data.

### Conflict of Interest

The authors declare that the research was conducted in the absence of any commercial or financial relationships that could be construed as a potential conflict of interest.
