# Supplementary figures and images for: CWPO Degradation of Methyl Orange at Circumneutral pH: Multi-Response Statistical Optimization, Main Intermediates and by-Products
Source: Front Chem. 2019 Nov 14;7:772. doi: 10.3389/fchem.2019.00772 (PMC6868118; doi:10.3389/fchem.2019.00772)

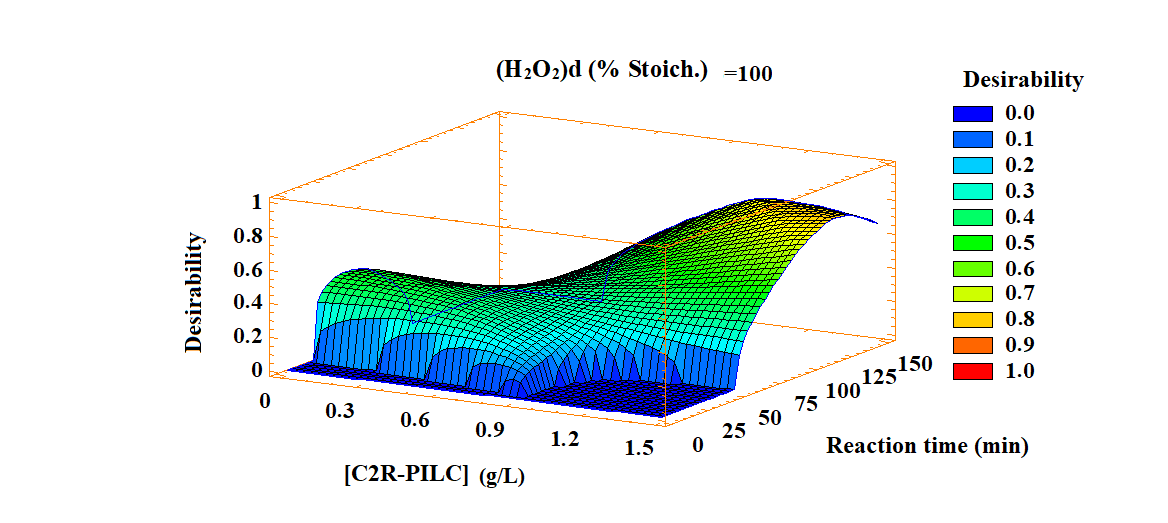

Supplement: Supplementary file 1 [file Image_1.TIF]

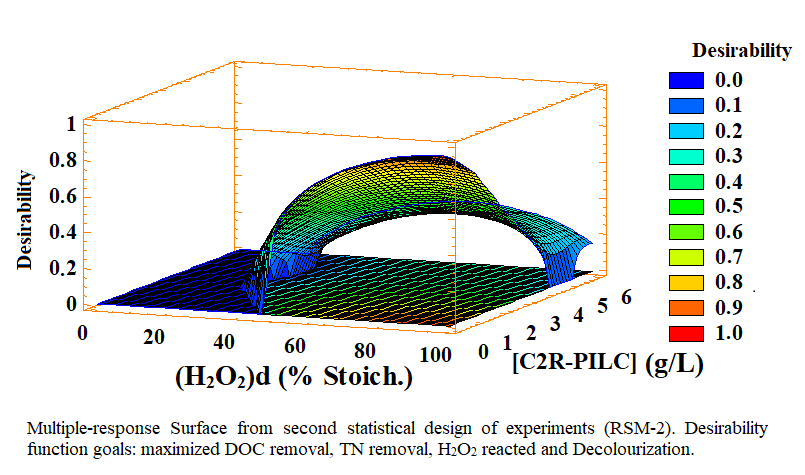

Supplement: Supplementary file 2 [file Image_2.TIF]

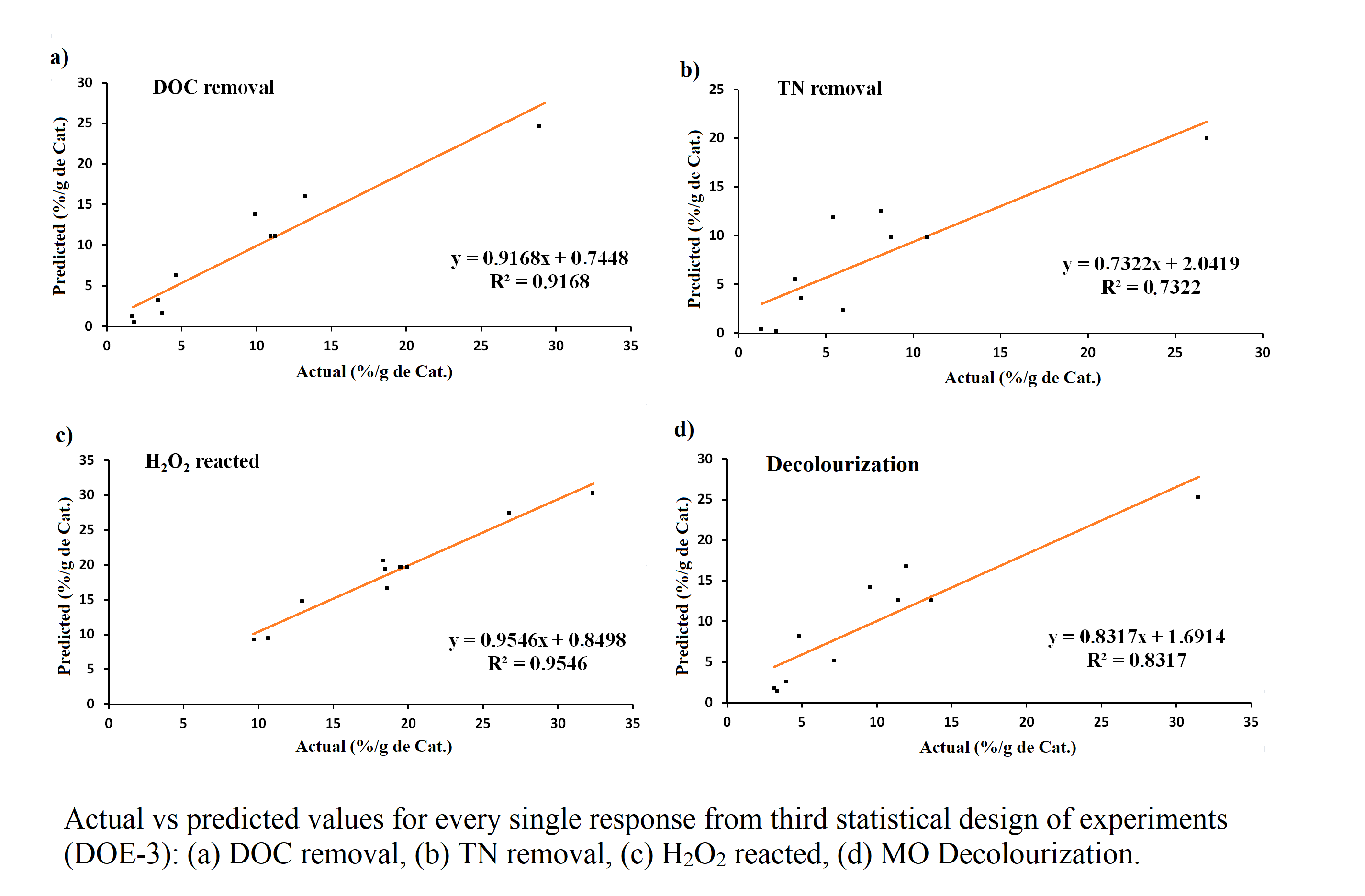

Supplement: Supplementary file 3 [file Image_3.TIF]

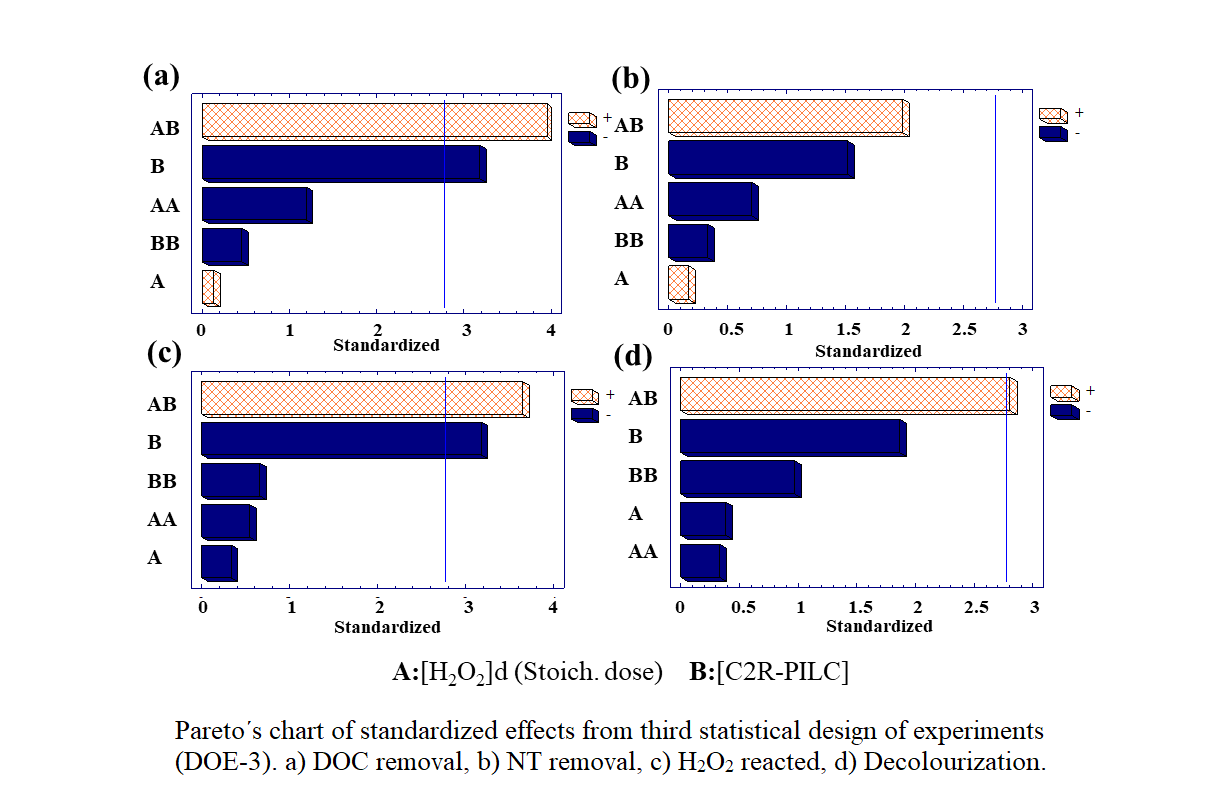

Supplement: Supplementary file 4 [file Image_4.TIF]
