# Supplementary material for: CWPO Degradation of Methyl Orange at Circumneutral pH: Multi-Response Statistical Optimization, Main Intermediates and by-Products
Source: Front Chem. 2019 Nov 14;7:772. doi: 10.3389/fchem.2019.00772 (PMC6868118; doi:10.3389/fchem.2019.00772)
Supplement: Supplementary file 5 [file Table_1.DOCX]

Physicochemical features of refined starting clay (C2R) and final clay catalyst (C2R-PILC).

| **Sample** | **Fe _Incorporated_ ^a^**  **(wt. %)** | ***d_001_ ^b^ (nm)*** | **S_BET_^c^ (m^2^/g)** | **S_µp_^d^**  **(m^2^/g)** | **S_Ext_^e^ (m^2^/g)** | **V_µp_^f^ (cm^3^/g)** | **Mean pore width (Å)** |
| --- | --- | --- | --- | --- | --- | --- | --- |
|  |  |  |  |  |  |  |  |
| C2R | NA | 1.50 | 91 | 36 | 55 | 0.0911 | 41.6 |
| C2R-PILC | 0.62 | 1.77 | 157 | 121 | 36 | 0.0997 | 29.3 |

NA: Not applicable; ^a^ Iron content incorporated in the pillared clays; ^b^ Obtained from powder samples; ^c^ S_BET_: specific surface area; ^d^ S_µp:_ surface of micropores; ^e^ S_Ext_: external surface; ^f^ V_µp_: Volume of micropores.
