# Supplementary material for: CWPO Degradation of Methyl Orange at Circumneutral pH: Multi-Response Statistical Optimization, Main Intermediates and by-Products
Source: Front Chem. 2019 Nov 14;7:772. doi: 10.3389/fchem.2019.00772 (PMC6868118; doi:10.3389/fchem.2019.00772)
Supplement: Supplementary file 6 [file Table_2.DOCX]

First statistical design of experiments (DOE-1) screening out main operating parameters of Al/Fe-PILC activated CWPO in the MO degradation

| Experimental Variables | Units | Lower level  (-1) | Upper level (+1) | central | Axial points |
| --- | --- | --- | --- | --- | --- |
| **(H_2_O_2_ )d** | Stoichiometric % | 50 | 150 | 100 | 15.9 – 184 |
| **[C2R-PILC]** | g/L | 0.34 | 1.21 | 0.78 | 0.05 – 1.50 |
| **Reaction time (tr)** | min | 60 | 120 | 90 | 39.5 – 140 |
| Covariates | | | | | |
| **Starting MO concentration ([MO]_i_)** | DOC (mg C/L) | 0 – 30 | | - | - |
| **Temperature of reaction (T)** | °C | 1.0 – 35 | | - | - |
| **pH** | - | 6.0 – 9.0 | | - | - |
