# Supplementary material for: CWPO Degradation of Methyl Orange at Circumneutral pH: Multi-Response Statistical Optimization, Main Intermediates and by-Products
Source: Front Chem. 2019 Nov 14;7:772. doi: 10.3389/fchem.2019.00772 (PMC6868118; doi:10.3389/fchem.2019.00772)
Supplement: Supplementary file 7 [file Table_3.DOCX]

Second statistical design of experiments (DOE-2) realizing more interesting levels for the experimental factors in the Al/Fe-PILC activated CWPO degradation of MO

| Experimental Variables | Units | Lower level  (-1) | Upper  Level  (+1) | central | Axial points |
| --- | --- | --- | --- | --- | --- |
| **(H_2_O_2_ )d** | Stoichiometric % | 36 | 89 | 62.5 | 25 – 100 |
| **[C2R-PILC]** | g/L | 1.30 | 5.20 | 3.25 | 0.5 – 6.0 |
| **Reaction time (tr)** | 180 minutes | | | | |
| Covariates | | | | | |
| **Starting MO concentration ([MO]_i_)** | DOC (mg C/L) | 2.0 – 20 | | - | - |
| **Temperature of reaction (T)** | °C | 5.0 – 25 | | - | - |
| **pH** | - | 6.0 – 9.0 | | - | - |
