# Supplementary material for: CWPO Degradation of Methyl Orange at Circumneutral pH: Multi-Response Statistical Optimization, Main Intermediates and by-Products
Source: Front Chem. 2019 Nov 14;7:772. doi: 10.3389/fchem.2019.00772 (PMC6868118; doi:10.3389/fchem.2019.00772)
Supplement: Supplementary file 8 [file Table_4.DOCX]

Experimental results from third statistical design of experiments (DOE-3) delivering optimized Al/Fe-PILC activated CWPO degradation of MO (responses expressed per g of clay catalyst)

| Run | Factor | | Responses^a^ | | | | (H_2_O_2_/Fe)  (mmol/mmol) | [Fe leached]^c^  (mg/L) | Fe leached^d^ (%) |
| --- | --- | --- | --- | --- | --- | --- | --- | --- | --- |
|  | **(H_2_O_2_)d**  **(% Stoich.)** | **[C2R-PILC] (g/L)** | **Mineralization** | | **Reacted H_2_O_2_ (%/g cat.)** | **Dec.^b^**  **(%/g cat.)** |  |  |  |
|  |  |  | **DOC removal**  **(%/g cat.)** | **TN**  **removal**  **(%/g cat.)** |  |  |  |  |  |
| 1 | 62.5 | 10.0 | 11 | 9.0 | 20 | 11 | 1.73 | 0.18 | 0.30 |
| 2 | 50.0 | 10.0 | 10 | 5.0 | 18 | 12 | 0.88 | 0.20 | 0.32 |
| 3 | 62.5 | 5.00 | 13 | 8.0 | 27 | 10 | 3.08 | 0.07 | 0.22 |
| 4 | 71.3 | 13.5 | 5.0 | 3.0 | 13 | 5.0 | 1.10 | 1.48 | 1.77 |
| 5 | 62.5 | 10.0 | 11 | 11 | 20 | 14 | 1.66 | 0.15 | 0.24 |
| 6 | 75.0 | 10.0 | 2.0 | 2.0 | 19 | 7.0 | 2.52 | 0.15 | 0.24 |
| 7 | 71.3 | 6.50 | 2.0 | 1.0 | 19 | 4.0 | 4.70 | 0.18 | 0.46 |
| 8 | 62.5 | 15.0 | 3.0 | 4.0 | 10 | 3.0 | 0.56 | 2.04 | 2.19 |
| 9 | 53.7 | 13.5 | 4.0 | 6.0 | 11 | 3.0 | 1.37 | 1.15 | 1.38 |
| 10 | 53.7 | 6.50 | 29 | 27 | 32 | 32 | 2.00 | 0.24 | 0.59 |

^a^ All expressed as (%/g cat.) to rule out adsorption from the statistical optimization; ^b^ Decolourization (466 nm); ^c^ Measured at final reaction time; ^d^ Respect to content of active Fe in the clay catalyst = 0.62 wt %.
