# Supplementary material for: CWPO Degradation of Methyl Orange at Circumneutral pH: Multi-Response Statistical Optimization, Main Intermediates and by-Products
Source: Front Chem. 2019 Nov 14;7:772. doi: 10.3389/fchem.2019.00772 (PMC6868118; doi:10.3389/fchem.2019.00772)
Supplement: Supplementary file 9 [file Table_5.DOCX]

Main intermediates and by-products measured along the CWPO degradation of MO under optimal reaction conditions

| Reaction time  (min) | MO Intermediates and by-products (mg/L) | | | | | | | | |
| --- | --- | --- | --- | --- | --- | --- | --- | --- | --- |
|  | **MO** | **PhO**^a^ | ***p*-BQ^b^** | **Oxálic**  **acid** | **AS^c^** | **PhA^d^** | **N-methyl aniline** | **N,N-DMA^e^** | **3-DAPhO^f^** |
| 0 | 98.9 | ND | ND | ND | ND | ND | ND | ND | ND |
| 30 | 73.6 | ND | ND | ND | ND | ND | ND | ND | ND |
| 75 | 56.4 | 3.14 | ^f^*Blq* | 9.89 | ND | 2.00 | 1.69 | 1.46 | ND |
| 120 | 45.9 | 7.22 | *Blq* | 14.7 | 3.15 | 1.48 | Blq | Blq | Blq |
| 165 | 40.7 | 4.30 | ND | 20.9 | 5.314 | ND | ND | ND | ND |
| 210 | 8.63 | 3.42 | ND | 43.8 | 7.71 | ND | ND | ND | ND |
| 240 | 6.98 | 1.11 | ND | 45.5 | 8.31 | ND | ND | ND | ND |

^a^ Phenol; ^b^ Cyclohexa-2,5-diene-1,4-dione; ^c^ 4-aminobencenzulfonic acid; ^d^ Phenylamine; ^e^ N,N-dimethylaniline; ^f^ 3-dimethylaminophenol; ND: Not detected; *Blq*: Below the limit of quantification.
